# Risk factors and outcomes after interruption of sedation in subarachnoid hemorrhage (ROUTINE-SAH)—a retrospective cohort study

**DOI:** 10.3389/fneur.2024.1363107

**Published:** 2024-03-13

**Authors:** Moritz L. Schmidbauer, Sebastian Läufer, Andreas Maskos, Konstantinos Dimitriadis

**Affiliations:** Department of Neurology, LMU University Hospital, LMU Munich, Munich, Germany

**Keywords:** SAH, sedation, interruption of sedation, ICP, neurocritical care

## Abstract

**Introduction:**

Aneurysmal subarachnoid hemorrhage (aSAH) often necessitates prolonged sedation to manage elevated intracranial pressure (ICP) and to prevent secondary brain injury. Optimal timing and biomarkers for predicting adverse events (AEs) during interruption of sedation (IS) after prolonged sedation are not well established. To guide sedation management in aSAH, we aimed to explore the frequency, risk factors, and outcomes of IS in aSAH.

**Methods:**

In a retrospective cohort study, a total of 148 patients with aSAH from January 2015 to April 2020 were screened. In total, 30 patients accounting for 42 IS were included in the analysis. Adverse events (AEs) during IS were used as core outcome measures and were categorized into neurological and non-neurological AEs. Baseline characteristics, clinical parameters before IS, AEs, and functional outcomes were collected using health records. Statistical analysis used generalized linear mixed-effects models with regularization to identify candidate predictors with subsequent bootstrapping to test model stability. As an exploratory analysis, multivariate linear and logistic regression was used to analyze the association between IS and intensive care unit length of stay, duration of mechanical ventilation, and functional outcomes.

**Results:**

The mean age was 56.9 (SD 14.8) years, and a majority of the patients presented with poor-grade SAH (16/30, 53.3%). Neurological and non-neurological AEs occurred in 60.0% (18/30) of the patients. Timing, number of IS attempts, ICP burden, craniectomy status, level of consciousness, heart rate, cerebral perfusion pressure, oxygen saturation, fraction of inspired oxygen, and temperature were selected as candidate predictors. Through bootstrapping, elapsed time since disease onset (OR 0.85, 95% confidence interval (95% CI) 0.75–0.97), ICP burden (OR 1.24, 95% CI 1.02–1.52), craniectomy (OR 0.68, 95% CI 0.48–0.69), and oxygen saturation (OR, 0.80 0.72–0.89) were revealed as relevant biomarkers for neurological AEs, while none of the pre-selected predictors was robustly associated with non-neurological AEs.

**Conclusion:**

In aSAH, complications during the definite withdrawal of sedation are frequent but can potentially be predicted using clinical parameters available at the bedside. Prospective multicenter studies are essential to validate these results and further investigate the impact of IS complications.

## 1 Introduction

Aneurysmal subarachnoid hemorrhage (aSAH) continues to be a major challenge with a high mortality rate and a substantial and lasting impact on functional outcomes and quality of life (1). As elevated intracranial pressure (ICP) is one hallmark of severe aSAH, prolonged sedation with subsequent reduction of cerebral metabolism, paralleled by a decline in cerebral blood volume and ultimately ICP, has been adopted as a therapeutic strategy to prevent further secondary brain injury (2). However, the paradox of sedation in neurocritical care—and aSAH in particular—is that over-sedation can be particularly harmful, as serial clinical neurological examinations are pivotal to allow timely management of common complications, such as re-bleeding or delayed cerebral ischemia (DCI) (3).

Based on data generated from randomized controlled trials involving the non-neurological intensive care population showing improved patient outcomes due to daily IS and spontaneous breathing trials (4, 5), smaller trials for brain-injured patients followed (6–9). In the latter case, however, trials consistently showed critical alterations in brain metabolism (6–8). As neurointensivists are therefore hesitant to adopt the practice of daily IS and spontaneous breathing trials, prolonged sedation is often only terminated after days in pathologies with relevant ICP elevation, such as aSAH. However, data regarding the optimal timing and clinical biomarkers predicting adverse events during IS after prolonged sedation are scarce, leading to uncertainty and heterogeneous management (10–12).

Hence, we aimed to describe the frequency, risk factors, and outcomes of IS in aSAH to guide awakening after prolonged sedation.

## 2 Methods

### 2.1 Study design and participants

**Figure 1:**
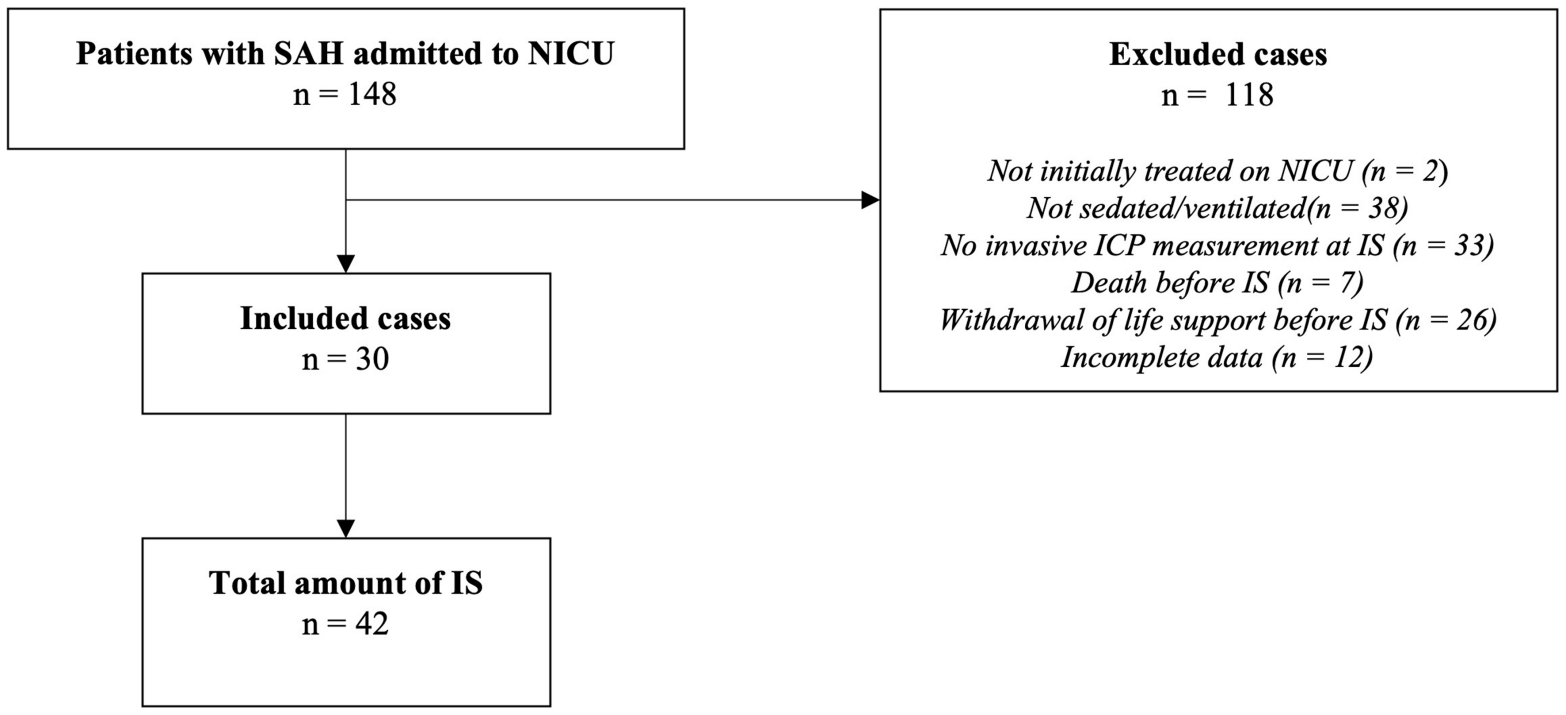
Study selection diagram. SAH, subarachnoid hemorrhage; NICU, neurointensive care unit; IS, interruption of sedation; ICP, intracranial pressure.

Following a retrospective design, patients with aSAH admitted to the Neurointensive Care Unit (NICU) of a tertiary academic center (LMU University Hospital, Munich, Germany) between January 2015 and April 2020 were screened (*n* = 148). Patients aged 18 years or older with sedation established at the time of ICU admission and invasive ICP monitoring within 24 h of ICU admission were included. Death or withdrawal of life support before the first IS led to the exclusion of the respective cases. Applying these criteria, data from 30 patients with a total of 42 IS were collected (Figure 1). The study was conducted in accordance with the Declaration of Helsinki and approved by the ethics committee of LMU Munich (protocol code 19-497, date of approval 07/30/2019).

### 2.2 Interruption of sedation (IS)

IS is defined as any attempt to significantly reduce or terminate sedation with the intention to awaken the patient. We used two mandatory criteria for IS: documentation in electronic health records (EHRs) of the plan to awaken the patient and the actual continuous reduction of sedatives (such as ketamine, propofol, midazolam, or isoflurane) occurring at least every 24 h. Timing and other clinical decisions surrounding IS were at the discretion of the treating physicians. Following local standards, analgosedation was routinely performed using propofol/sufentanil (<7 days). In cases necessitating longer sedation, the regimen was switched to ketamine/midazolam or isoflurane. As opioids are not adequate to induce sedation for neuroprotection in acute brain injury, a reduction in the opioid dose did not qualify as IS for this study. IS was considered as failed if either a bolus of sedative was applied more than twice within 6 h or the continuous infusion rate was re-raised without a subsequent reduction to baseline within 6 h.

### 2.3 Data collection

Data on baseline parameters, characteristics of IS, and outcome parameters were systematically extracted from patient charts and EHRs.

Predictor variables included elapsed time since disease onset, number of previous IS, hours with ICP >20 mmHg (burden of ICP, within 24 prior to IS), as well as the following clinical parameters as recorded immediately before IS: heart rate, systolic blood pressure, diastolic blood pressure, mean arterial pressure, oxygen saturation (SpO2), body temperature, cerebral perfusion pressure (CPP), intracranial pressure (ICP), Glasgow Coma Scale (GCS), fraction of inspired oxygen (FiO2), positive end-expiratory pressure (PEEP), Richmond Agitation Sedation Scale (RASS) above -5, and noradrenaline dosage. Furthermore, the last available imaging before IS was screened for radiological evidence of a focal space-occupying lesion, midline shift >5 mm, or global cerebral edema (GCE) as previously defined (13).

Thresholds of adverse events (AEs) as outcome measures were chosen in accordance with the previous literature on awakening trials in SAH (ICP > 20 mmHg, CPP < 50 mmHg for >5 min, respiratory rate 35 for >5 min, oxygen saturation (SpO2) < 88% for >5 min, signs of distress [tachycardia or bradycardia (>130/min; <50/min), diaphoresis, abdominal paradox, marked dyspnea, or use of accessory muscles)] (6). Additionally, a binary radiological endpoint was added to evaluate the presence of GCE, re-bleeding, and/or progressive local swelling on cranial imaging after IS. To evaluate the potential risk factors associated with AEs, we developed composite outcome metrics for neurological and non-neurological endpoints. Specifically, the endpoint of neurological AEs was considered positive if ICP, CPP, or established radiological thresholds were exceeded. Similarly, non-neurological AEs were identified if respiratory rate, SpO2, or predefined distress thresholds were surpassed.

### 2.4 Statistical analysis

Data were characterized using means ± standard deviations (SD) for continuous variables and medians with interquartile ranges (IQR) for categorical variables. In addition, binary variables were depicted as frequencies. To compensate for missing entries, predictive mean matching was employed, accounting for 3.2% of data imputation. Multicollinearity among predictors was mitigated by excluding variables with a variance inflation factor (VIF) exceeding 5 (14) (systolic blood pressure prior to IS, diastolic blood pressure prior to IS, and mean arterial pressure prior to IS). The relationship between predictors and outcomes was initially explored through scatter plots, confirming the absence of any non-linear dependencies. Due to patients experiencing multiple IS and thus contributing repeated measurements, we employed a generalized linear mixed-effects model (GLMM) with individuals as random effects to properly account for the clustered structure of the data. The challenge of a low event-to-predictor ratio was managed by applying least absolute shrinkage and selection operator (LASSO) regularization to refine the model by excluding variables that did not contribute to the prediction of neurological AEs. Notably, factors associated with individual patients such as baseline characteristics were therefore not modeled as separate independent variables. To assure comparability between the different units of the predictors, standardization was conducted (standard deviation units) for the GLMM-LASSO model. For example, an odds ratio of 2 thus means that—holding all other independent variables constant—a 1 SD increase in the predictor variable doubles the odds of the occurring outcome. Hence, units of the respective variables become irrelevant. Model optimization involved a 10-fold cross-validation to select the most appropriate lambda value, followed by the residual analysis to evaluate model fit (For model diagnostics, please refer to Supplementary Figure 1). As LASSO does not provide any statistical inference, we applied bootstrapping with 1,000 iterations to construct a sampling distribution of the LASSO model and to generate confidence intervals as a measure of uncertainty (15). To enhance interpretability, regression coefficients were converted to odds ratios (ORs). Furthermore, as an exploratory analysis, the impact of the total number of IS and the number of IS with AEs on functional outcomes at discharge from the hospital, discharge from the rehabilitation unit, ICU length of stay (ICU LOS), and duration of mechanical ventilation was analyzed via multivariate regression models adjusted for age and WFNS. Statistical analyses and visualizations were performed using R (version 2023.06.1 + 524) with the glmmLasso, ggplot2, car, lme4, pROC, boot, and dplyr packages. ChatGPT version 4.5 was used for error handling, repetitive programming, and overall optimization of code in R.

## 3 Results

### 3.1 Baseline parameters and outcomes after IS

Table 1 depicts the demographic characteristics of the SAH cohort. Overall, the patients had a mean age of 56.9 years (SD 14.8) and were predominantly female (18/30, 60.0%). Furthermore, approximately half of the patients presented with poor-grade SAH (WFNS 4–5) (16/30, 53.3%), 70% (21/30) showed anterior circulation aneurysms, and 83.3% (25/30) were treated via endovascular intervention. ICU length of stay was 32.0 days (SD 14.8) on average. Upon discharge from rehabilitation, the median mRS was 3.0 (IQR 1.3–4.0). Neurological and non-neurological AEs occurred in 60.0% (18/30) of the patients (Table 2).

**Table 1:** Baseline parameters.

Age, mean (SD)		56.9 (14.8)
Female sex, n (%)		18/30 (60.0)
Number of IS per patient, mean (SD)		1.7 (0.9)
GCS at admission, median (IQR)		8.5 (3.3–15.0)
FOUR score at admission, median (IQR)		13.0 (4.0–16.0)
WFNS	1	10/30 (33.3)
	2	2/30 (6.7)
	3	2/30 (6.7)
	4	3/30 (10.0)
	5	13/30 (43.3)
Modified Fisher Scale	1	–
	2	1/30 (3.3)
	3	5/30 (16.7)
	4	24/30 (80.0)
Sentinel bleeding, n (%)		11/30 (36.7)
Seizure at onset, n (%)		9/30 (30.0)
Secondary aneurysms, n (%)		8/30 (26.7)
Herniation at admission, n (%)		8/30 (26.7)
Acute hydrocephalus at admission, n (%)		17/30 (56.7)
Midline shift >5 mm at admission, n (%)		7/30 (23.3)
Aneurysm treatment, n (%)	Endovascular	25/30 (83.3)
	Surgical	5/30 (16.7)
Aneurysm location, n (%)	Anterior circulation	21/30 (70.0)
	Posterior circulation	9/30 (30.0)
SAPS II at admission, median (IQR)		38.5 (34.3–49.0)

SD, standard deviation; GCS, Glasgow Coma Scale; IQR, interquartile range; FOUR, Full Outline of UnResponsiveness; WFNS, World Federation of Neurosurgical Societies; LoC, loss of consciousness; SAPS II, Simplified Acute Physiology Score II; MV, mechanical ventilation; ICU LOS, intensive care unit length of stay; mRS, modified Rankin Scale.

**Table 2:** Outcomes after IS.

Number of IS aborted by treating NICU team, n (%)	11/42 (26.0)
Duration of MV, mean (SD)	19.5 (14.4)
ICU LOS, mean (SD)	32.0 (14.8)
mRS at hospital discharge, median (IQR)	5.0 (4.0–5.0)
mRS at discharge from rehabilitation, median (IQR)	3.0 (1.3–4.0)
Neurological AEs, n (%)	18/30 (60.0)
Non-neurological AEs, n (%)	18/30 (60.0)

IS, interruption of sedation; NICU, neurointensive care unit; MV, mechanical ventilation; SD, standard deviation; IQR, interquartile range; ICU LOS, intensive care unit length of stay; mRS, modified Rankin Scale; AEs, adverse events.

### 3.2 Timing of IS

Through stratification by SAH severity (good-grade SAH, WFNS 1–3 versus poor-grade SAH, WFNS 4–5), distinct patterns in the timing of IS are revealed. Specifically, most IS in good-grade SAH occur early within the first 5 days after disease onset (3.5 ± 3.6), whereas IS in poor-grade SAH peaks at approximately 10 days after ictus (11.6 ± 5.1) (Supplementary Figure 2).

### 3.3 Risk factors for AEs during IS

**Figure 2:**
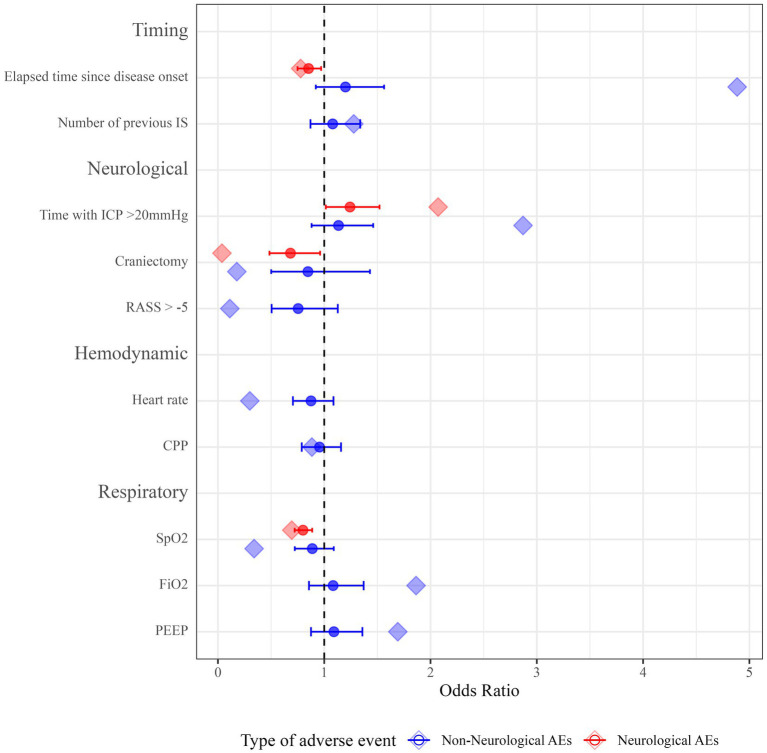
Odds ratios for predictor variables of neurological and non-neurological AEs. Point estimates for ORs from the GLMM-LASSO model are represented as diamond-shaped data points. ORs from bootstrapping are represented as circles. Horizontal lines represent 95% CIs after applying the model to the bootstrapping sample. IS, interruption of sedation; ICP, intracranial pressure; RASS, Richmond Agitation Sedation Scale; bpm, beats per minute; CPP, cerebral perfusion pressure; SpO2, oxygen saturation; FiO2, fraction of inspired oxygen; PEEP, positive end-expiratory pressure; 95% CI, 95% confidence interval; ORs, odds ratios; GLMM-LASSO, generalized linear mixed-effects models with least absolute shrinkage and selection operator; 95% CIs, 95% confidence intervals.

Descriptive statistics for all predictor variables are provided in Supplementary Table 1. For neurological AEs during IS, time since disease onset, ICP burden, oxygen saturation, and craniectomy status were selected as candidate predictor variables via the GLMM-LASSO model (Figure 2 and Supplementary Table 2). When applied to a bootstrapping sample, all variables showed robust results (time since disease onset—OR 0.85, 95% confidence interval (95% CI) 0.75–0.97; ICP burden—OR 1.24, 95% CI 1.02–1.52; craniectomy—OR 0.68, 95% CI 0.48–0.69; oxygen saturation—OR 0.80, 95% CI 0.72–0.89). Conclusively, higher values for elapsed time and oxygen saturation as well as craniectomy are protective for neurological AEs, while higher ICP burden increases the likelihood of neurological AEs (Figure 2 and Supplementary Table 3).

For non-neurological AEs during IS, time since disease onset, number of previous IS, ICP burden, craniectomy status, level of consciousness, heart rate, CPP, oxygen saturation, FiO2, and PEEP were selected as potential predictors (Figure 2 and Supplementary Table 2). However, after running a bootstrapping sample on this model, none of the selected variables was significantly associated with non-neurological AEs (Figure 2 and Supplementary Table 3).

### 3.4 Impact of adverse events during IS on clinical endpoints

With multivariate regression, adjusting for WFNS and age, the total number of IS attempts per patient and the number of IS attempts with AEs were not independently associated with worse functional outcomes (mRS 3–6) at hospital discharge (OR 1.8, 95% CI 0.05–68.3, *p* = 0.73/OR 1.9, 95% CI 0.2–22.0, *p* = 0.62) and discharge from rehabilitation (OR 0.8, 95% CI 0.2–3.0, *p* = 0.75/1.0, 95% CI 0.3–3.3, *p* = 0.94), respectively. Moreover, ICU length of stay (regression coefficient (beta) −2.0, 95% CI −9.0–5.1, *p* = 0.59/beta 0.3, 95% CI −5.6–6.1, *p* = 0.93) and duration of mechanical ventilation (beta 0.8, 95% CI −7.3–8.8, *p* = 0.85/ beta 2.6, 95% CI −3.9–9.2, *p* = 0.44) also showed no significant association (Table 3). However, broad confidence intervals suggest considerable uncertainty around these estimates.

**Table 3:** Multivariate regression models for functional outcomes, ICU LOS, and duration of mechanical ventilation.

	mRS 3–6 at hospital discharge	mRS 3–6 at discharge from rehabilitation	ICU LOS	Duration of MV
Number of IS attempts, total	1.8 (0.05–68.3), *p* = 0.73	0.8 (0.2–3.0), *p* = 0.75	−2.0 (−9.0–5.1), *p* = 0.59	0.8 (−7.3–8.8), *p* = 0.85
Number of IS attempts with AEs	1.9 (0.2–22.0), *p* = 0.62	1.0 (0.3–3.3), *p* = 0.94	0.3 (−5.6–6.1), *p* = 0.93	2.6 (−3.9–9.2), *p* = 0.44

OR with 95% CI and corresponding *p*-values are depicted for functional outcomes at hospital discharge and discharge from rehabilitation. For ICU LOS and duration of MV, regression coefficients with 95% CI and corresponding *p*-values are provided.

IS, interruption of sedation; mRS, modified Rankin Scale; ICU, intensive care unit; LOS, length of stay; MV, mechanical ventilation.

## 4 Discussion

In this cohort study, we present a thorough analysis of the frequency, risk factors, and outcomes associated with IS in patients with aSAH. The main results are as follows: (i) the timepoint of definite withdrawal of sedation was associated with aSAH severity and peaked within 5 days for good-grade aSAH and 10 days for poor-grade aSAH; (ii) AEs in the context of IS occurred in 60% of the patients; (iii) higher values for elapsed time and oxygen saturation as well as craniectomy are protective for neurological AEs, while higher ICP burden increases the likelihood of neurological AEs; and (iv) in an exploratory analysis, IS attempts were not associated with functional outcomes, duration of mechanical ventilation, and ICU length of stay.

In a cohort with brain injury of diverse etiology and similar thresholds for AEs, Helbok et al. found complications in one-third of the cohort during routine (daily) wake-up trials (6). While Helbok et al. reported a sedation-free median time of 35 min and looked at daily wake-up trials, our exposure of interest was the definite withdrawal of sedation. Accordingly, we observed the entire interval of sedation weaning, which might explain the higher frequency of AEs in our cohort. Given the broad confidence intervals and low sample size of our exploratory analysis, coupled with a 60% complication rate during IS documented in our data, there is an urgent need for prospective trials to further explore the clinical implications of AEs during IS.

Two larger surveys on sedation protocols in the neurointensive care population showing an association between aSAH severity and timing of IS support our data (10, 11). In one study, the duration of prolonged sedation for patients with unfavorable biomarkers, such as therapy refractory ICP or radiological surrogates for elevated ICP, was reported with a mean of 4.5 days (SD 1.8) for good-grade SAH and 5.6 days (SD 2.8) for poor-grade SAH (10). Given the limited data on the effectiveness of prolonged sedation and the optimal timing for IS, alongside the correlation of ICP elevation with SAH severity and disease duration, there is a compelling pathophysiological basis to tailor sedation duration and intensity based on WFNS grading and time since disease onset (16–18). This is in line with our analysis showing lower odds of neurological AEs with increasing time since disease onset (OR 0.85, 95% CI 0.75–0.97). While the higher odds of non-neurological AEs with longer sedation are not significant in our data, prolonged mechanical ventilation without spontaneous breathing and high amounts of sedatives are known to be harmful in the general ICU population as shown by several landmark trials almost two decades ago (4, 5). The paradox of sedation in neurointensive care—beneficial for neurological parameters yet potentially harmful to other organ systems—emphasizes the need for more data to allow for accurate dosing and timing.

In our study, we identified the ICP burden (OR 1.24, 95% CI 1.02–1.52), but not baseline ICP before IS, as a risk factor for neurological AEs. This finding aligns with the emerging evidence suggesting that cumulative ICP exposure, represented as the area under the curve in an ICP–time diagram, is a more reliable prognostic biomarker than single ICP measurements at arbitrary timepoints in acute brain injury (17, 19). The finding that ICP burden over the course of the disease is a predictor of future neurological AEs is further supported by a published machine learning algorithm showing altered ICP as long as 24 h before the crisis predicts future surges in ICP (20, 21). Similarly, the presence of decompressive craniectomy before IS was beneficial for neurological AEs, most likely as a surrogate for lower ICP during IS.

Unlike the neurological endpoints, the chosen predictors for non-neurological AEs failed to hold up under bootstrapping analysis. The observed heterogeneity might be explained by the broad spectrum covered by this composite outcome—encompassing respiratory events, deviations in heart rate, and agitation—each potentially stemming from distinct pathophysiological conditions not adequately captured by the predictors.

There are several limitations in this study. First, biases inherent to retrospective studies apply to our study as well. In this context, performance bias, with different handling of IS according to the risk assumed by the healthcare providers, might be particularly relevant. Second, the sample size is small, limiting the robustness of the results. Thus, despite limiting the number of variables via regularization with LASSO, the models on risk factors for AEs during IS might be overfitted. For the same reason, the adjustment of multivariate regression models for confounders other than WFNS and age was not appropriate, which potentially could introduce unmeasured confounding. The small sample size also makes it difficult to detect the effect of IS complications on functional outcomes or ICU treatment characteristics. Moreover, outcome data beyond discharge from rehabilitation are lacking. Third, the local practices of the study center in a monocentric setting will greatly influence the selection of predictor variables and their effect size. The strengths are the utilization of a multistep statistical analysis to account for the data structure and the appreciation of different pathophysiologies of non-neurological and neurological AEs by reporting these endpoints and their predictors separately. In addition, bootstrapping was used to compensate for the low sample size and to provide more robust estimates including measures of uncertainty. Overall, to the best of our knowledge, this is the first study looking into the specific predictors of complications in the context of IS in aSAH.

## 5 Conclusion

In aSAH patients, complications during the definite withdrawal of sedation are frequent. Neurological AEs during IS can potentially be predicted using time since disease onset, ICP burden, craniectomy status, and oxygen saturation. Prospective multicenter studies are warranted to validate these results and further investigate the impact of AEs during IS on functional outcomes.

## Data availability statement

The original contributions presented in the study are included in the article/Supplementary material, further inquiries can be directed to the corresponding author.

## Ethics statement

The studies involving humans were approved by Ethikkommission LMU München (20-647). The studies were conducted in accordance with the local legislation and institutional requirements. The ethics committee/institutional review board waived the requirement of written informed consent for participation from the participants or the participants’ legal guardians/next of kin due to the retrospective and anonymised nature of the study.

## Author contributions

MS: Conceptualization, Data curation, Formal analysis, Funding acquisition, Investigation, Methodology, Project administration, Resources, Software, Visualization, Writing – original draft. SL: Data curation, Investigation, Writing – review & editing. AM: Formal analysis, Methodology, Software, Writing – review & editing. KD: Conceptualization, Methodology, Project administration, Resources, Supervision, Validation, Writing – review & editing.
