# Supplementary material for: Risk factors and outcomes after interruption of sedation in subarachnoid hemorrhage (ROUTINE-SAH)—a retrospective cohort study
Source: Front Neurol. 2024 Mar 13;15:1363107. doi: 10.3389/fneur.2024.1363107 (PMC10965800; doi:10.3389/fneur.2024.1363107)
Supplement: Supplementary file 1 [file Data_Sheet_1.pdf]

Supplementary Material

1.1 Supplemental Tables

Elapsed time since disease [days] onset, mean (SD)	9.2 (6.3)
Number of previous IS, mean (SD)	1.4 (0.7)
Time with ICP >20mmHg [mmHg], mean (SD)	0.8 (1.9)
Heart rate [bpm], mean (SD)	76.7 (15.4)
SpO2 [%], mean (SD)	97.9 (1.4)
Temperature [° Celsius], mean (SD)	36.9 (1.4)
CPP [mmHg], mean (SD)	81.2 (13.6)
Global cerebral edema, n (%)	3/30 (10.0)
Craniectomy, n (%)	5/30 (16.7)
FiO2 [%], mean (SD)	39.8 (9.8)
PEEP [mbar], mean (SD)	7.2 (2.1)
RASS > -5, n (%)	8/30 (26.7)
Noradrenalin [mg/h], mean (SD)	0.5 (0.7)

Supplemental Table 1.
Descriptive statistics for predictor variables
Variables represent the values immediately before IS was commenced.
SD – standard deviation, ICP – intracranial pressure, SpO2 – oxygen saturation, CPP- cerebral perfusion pressure, FiO2 – fraction of inspired oxygen, PEEP – positive end expiratory pressure, RASS – Richmond Agitation Sedation Scale

**Variable**	**OR non-neurological AEs**	**OR neurological AEs**
Elapsed time since disease onset	4.88	0.78
Number of previous IS	1.28	-
Time with ICP >20mmHg	2.87	2.07
Heart rate	0.30	-
SpO2	0.34	0.69
Temperature	-	-
CPP	0.88	-
Global cerebral edema	-	-
Craniectomy	0.18	0.04
FiO2	1.86	-
PEEP	1.69	-
RASS > -5	0.11	-
Noradrenalin dosage	-	-

Supplemental Table 2.
Odds ratios of logistic regression with LASSO regularization

IS – interruption of sedation, ICP – intracranial pressure, RASS – Richmond Agigitation Sedation Scale, CPP – cerebral perfusion pressure, SpO2 – oxygen saturation, FiO2 – fraction of inspired oxygen, PEEP – positive end expiratory pressure

**Variable**	**OR (95% CI) non-neurological AEs**	**OR (95% CI) neurological AEs**
Elapsed time since disease onset	1.20 (0.92-1.56)	0.85 (0.75-0.97)
Number of previous IS	1.08 (0.87-1.34)	-
Time with ICP >20mmHg	1.13 (0.88-1.46)	1.24 (1.02-1.52)
Heart rate	0.88 (0.71-1.09)	-
SpO2	0.89 (0.72-1.09)	0.80 (0.72-0.89)
Temperature	-	-
CPP	0.96 (0.79-1.16)	-
Global cerebral edema	-	-
Craniectomy	0.85 (0.50-1.4)	0.68 (0.48-0.69)
FiO2	1.08 (0.86-1.37)	-
PEEP	1.09 (0.88-1.36)	-
RASS > -5	0.76 (0.51-1.13)	-
Noradrenalin dosage	-	-

Supplemental Table 3.
Odds ratios with 95% confidence intervals on bootstrapping sample
IS – interruption of sedation, ICP – intracranial pressure, RASS – Richmond Agigitation Sedation Scale, CPP – cerebral perfusion pressure, SpO2 – oxygen saturation, FiO2 – fraction of inspired oxygen, PEEP – positive end expiratory pressure

1.2 Supplementary Figures

A **B**

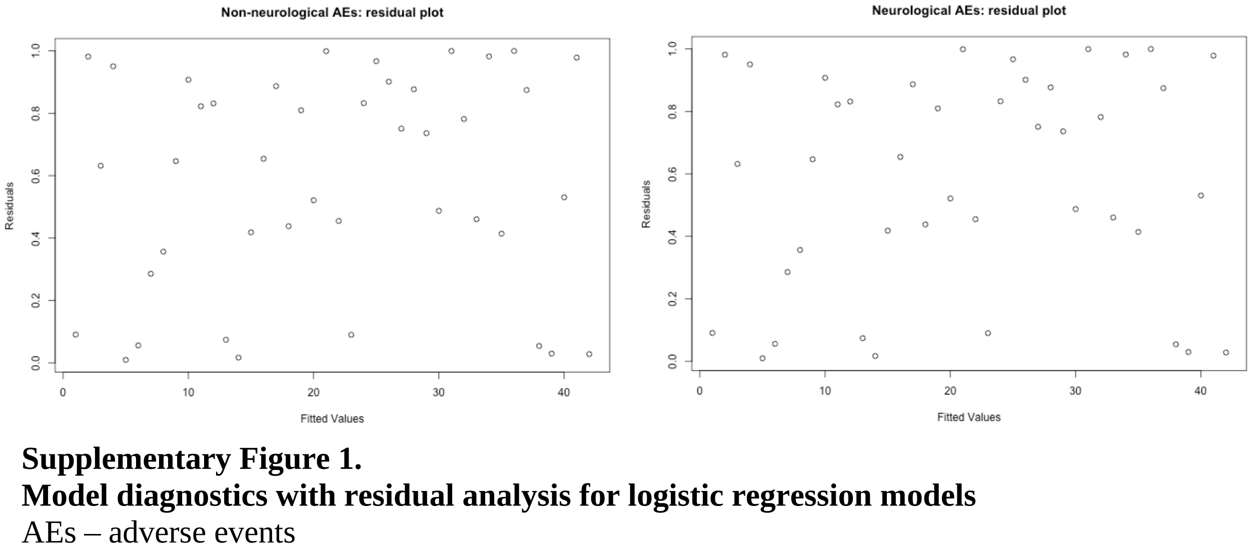

Supplementary Figure 1.
Model diagnostics with residual analysis for logistic regression models
AEs – adverse events
