# Supplementary figures and images for: Risk factors and outcomes after interruption of sedation in subarachnoid hemorrhage (ROUTINE-SAH)—a retrospective cohort study
Source: Front Neurol. 2024 Mar 13;15:1363107. doi: 10.3389/fneur.2024.1363107 (PMC10965800; doi:10.3389/fneur.2024.1363107)

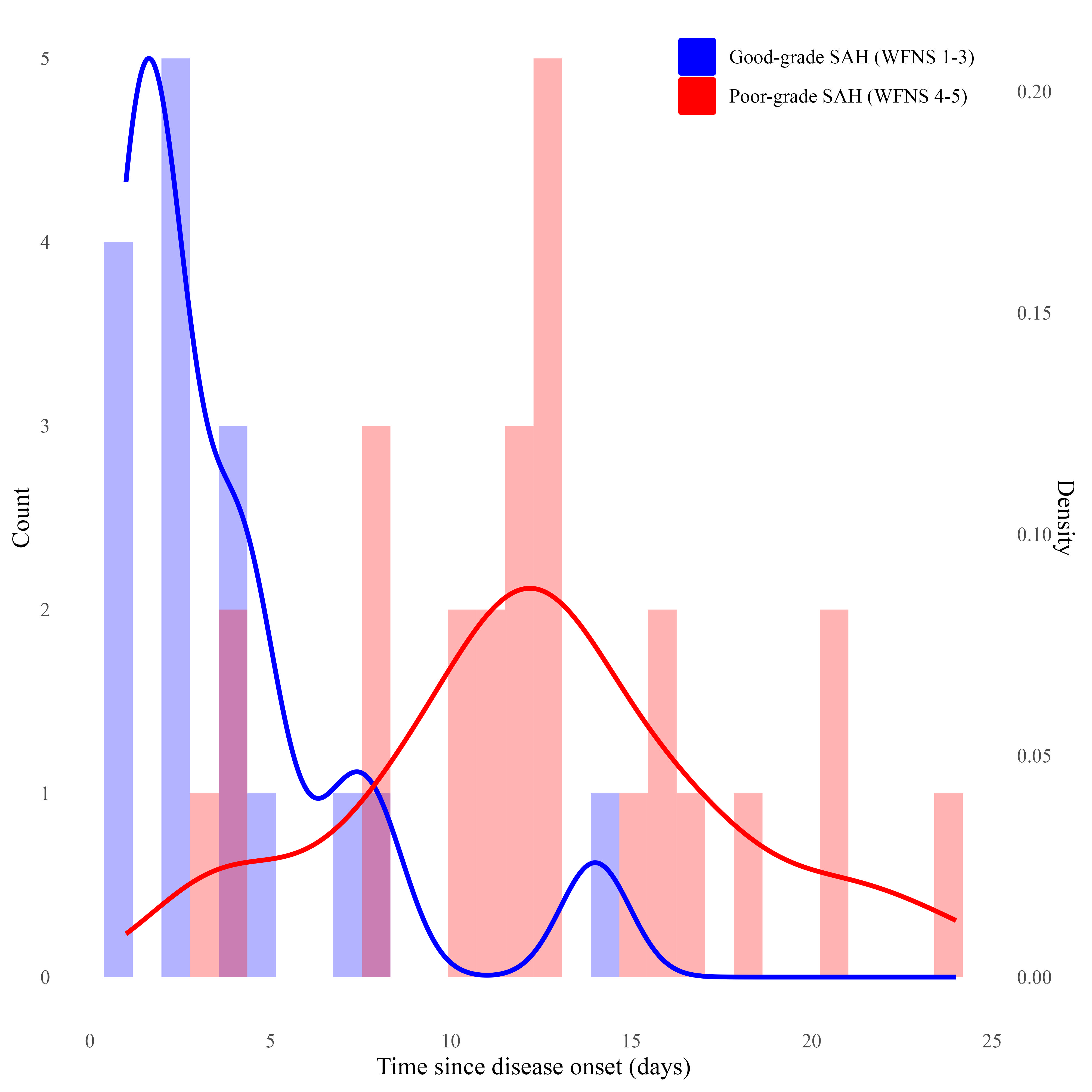

Supplement: Supplementary file 2 [file Image_2.jpeg]
